# Sorafenib induces ferroptosis in human renal cell carcinoma cells through CCAT/enhancer-binding protein homologous protein

**DOI:** 10.1016/j.bbrep.2025.102143

**Published:** 2025-07-07

**Authors:** Dinh Thi Nhung, Obadah E.A. Yousif, Byungsuk Kwon

**Affiliations:** a School of Biomedical Health Science and Engineering, Ulsan, 44610, Republic of Korea; b Graduate School of Biomedical Sciences, University of Ulsan, Ulsan, 44610, Republic of Korea

**Keywords:** Sorafenib, Renal cell carcinoma, ER stress, Ferroptosis, CHOP

## Abstract

Sorafenib, a multi-kinase inhibitor, has been shown to induce ferroptosis, a form of lipid peroxidation-mediated cell death. However, a mechanism of how sorafenib-induced ER stress leads to ferroptosis remains unclear. Here, we report that the CCAT/enhancer-binding protein (C/EBP) homologous protein (CHOP) is a critical mediator linking ER stress to ferroptosis in human renal cell carcinoma (RCC) cells after exposure to sorafenib. A large portion of sorafenib-induced cell death was shown to be caused by ferroptosis and ER stress significantly contributed to this ferroptic cell death. Among three major ER stress pathways, sorafenib specifically induced activation of the ATF4-CHOP axis. CHOP in turn functioned as an effector suppressing expression of SLC7A11. Therefore, our results suggest that sorafenib induces ferroptosis in RCC cells by increasing uncontrolled oxidative stress.

## Highlights

• Sorafenib induces ER stress in RCC cells through activation of the ATF4-CHOP axis.
• CHOP is required for sorafenib-induced ferroptosis in RCC cells.
• CHOP suppresses expression of SLC7A11 involved in cystine uptake.

## 1 Introduction

Sorafenib is an oral multikinase inhibitor drug targeting both serine/threonine and tyrosine kinases and it has been approved for the treatment of hepatocellular carcinoma, renal cell carcinoma (RCC), and differentiated thyroid carcinoma [[1], [2], [3]]. Despite the emergence of newer therapeutic agents, sorafenib remains a foundational treatment option for these malignancies. In addition to its established cytotoxic mechanisms through inhibition of tumor cell proliferation and angiogenesis, accumulating evidence suggests that sorafenib can also induce ferroptosis [4] - a novel form of regulated cell death characterized by iron-dependent lipid peroxidation and oxidative stress.

Ferroptosis is defined by the lethal accumulation of lipid peroxides and redox imbalance [5], often resulting from the suppression of intracellular antioxidant defense systems such as the Xc^−^ cystine/glutamate antiporter system (with SLC7A11 as its key transporter subunit) [6,7] and the lipid peroxide-detoxifying enzyme glutathione peroxidase 4 [8]. Several studies have demonstrated that sorafenib may inhibit SLC7A11, thereby reducing cystine uptake and sensitizing cancer cells to oxidative stress [[9], [10], [11]]. However, the intracellular signaling pathways that regulate sorafenib-induced ferroptosis remain incompletely understood in RCC.

Recent studies have highlighted the role of endoplasmic reticulum (ER) stress as a critical contributor to sorafenib-induced ferroptosis [12]. The ER is responsible for proper protein folding and post-translational modifications; when its folding capacity is overwhelmed, it initiates the unfolded protein response (UPR) to restore cellular homeostasis [13,14]. However, prolonged or excessive ER stress can lead to cellular dysfunction and cell death through apoptosis, necrosis, or ferroptosis [15]. The UPR is mediated by three main sensors: inositol-requiring enzyme 1 (IRE1) that activates the transcription factor X-box binding protein 1 (XPB1) through mRNA splicing, activating transcription factor 6 (ATF6), and protein kinase RNA-like ER kinase (PERK). Among these, the PERK-ATF4 signaling axis has been particularly associated with the transcriptional activation of C/EBP homologous protein (CHOP), a pivotal transcription factor in ER stress-induced cell death pathways.

CHOP plays a dual role in promoting both apoptosis and ferroptosis. It does so by regulating the expression of pro-apoptotic genes such as Bim and by enhancing oxidative stress through the suppression of antioxidant responses [16]. Recent studies have shown that CHOP is not merely a marker of ER stress but also a key mediator of ferroptosis in cancer cells undergoing chemotherapy or radiotherapy, including sorafenib-based treatments [17]. In this study, we provide evidence that sorafenib enhances ferroptosis by suppressing expression of SLC7A11 via CHOP in RCC cells. CHOP's ability to promote lipid peroxide accumulation and disrupt iron homeostasis may place it at the center of the link between ER stress and ferroptotic cell death.

## 2 Material and methods

### 2.1 Reagents

Sorafenib was purchased from Sigma-Aldrich. RSL3, Ferrostatin-1, and 4-1 phenylbutyrate (4-PBA) were purchased from Medchem-Express.

### 2.2 Cell line and cell culture

A human A498 RCC cell line was purchased from the Korean Cell Line Bank. A498 cells were cultured in Dulbecco's modified eagle's medium (DMEM) (Wellgene, Korea), 10 % fetal bovine serum (FBS) (Wellgene, Korea), and 10 % Zell shield. Cells were maintained in a 37 °C incubator with 5 % CO_2_.

### 2.3 CCK8 assay

Cell viability was assessed using the Cell Counting Kit-8 (CCK8, Cat. No. CK04) (Dojindo) following the manufacturer's instructions. Cells were seeded in 96-well plates at a density of 5000 cells per well and allowed to adhere for at least 12 h. Cells were then treated with the indicated compounds for the specified time periods. Then, 10 μL of CCK-8 reagent was added to each well and incubated for 2 h at 37 °C in a 5 % CO_2_ incubator. Absorbance was measured at 450 nm using a SpectraMax ID3 microplate reader (Molecular Devices). Cell viability was expressed as a percentage relative to the control group.

### 2.4 Real-time (RT)-PCR analysis

Total RNA was purified from cells using 1 mL of EasyBlue and 200 μL of chloroform, and the RNA layer was separated using a centrifuge. Synthesis of cDNA was performed according to the RT-PCR manufacturer's instruction (Invitrogen). RT-PCR was performed using 2x Amfisure Q-PCR Master Mix (GenoDEPOT). Triplicate samples per condition were analyzed and differences in gene expression compared to GAPDH internal reference control were calculated using the ΔΔCt method with the control group set to 1 [18]. The ΔΔCt method (also known as the comparative Ct method) calculates relative gene expression by comparing the Ct (threshold cycle) values of target genes normalized to an internal control (GAPDH) and relative to a calibrator (the control group). The formula used was: ΔCt = Ct (target gene) – Ct (GAPDH), ΔΔCt = ΔCt (treated sample) – ΔCt (control sample), and the fold change in expression = 2^(−ΔΔCt)^. Primer pairs for the target genes were as follows:

GRP78: forward, 5′-CACCTATTCCTGCGTCGGT-3′ and reverse, 5′-GTGAAGGCCACATACGACGG-3′; ATF4: forward, 5′-ATGAGCTTCCTGAACAGCGAA-3′ and reverse, 5′-CTTGTCGCTGGAGAACCCAT-3′; ATF6: forward, 5′-AAGCATAATCCGATCAGGCA-3′ and reverse, 5′-AAGGAGGTGCGTTGAAAACAA-3′; XPB1: forward 5′- TGCTGAGTCCGCAGCAGGTG-3′ and reverse, 5′-GCTGGCAGGCTCTGGGGAAG-3; CHOP: forward, 5′-AGCCAAAATCAGAGCTGGAA-3′ and reverse, 5′-TGG ATCAGTCTGGAAAAGCA-3; SLC7A11: forward, 5′- GAAGCATTCCCAGGGGCTAA-3′ and reverse, 5′-ACAGTGACGTTACAAACACCG-3′.

PCR products were separated on 2 % agarose gels stained with ethidium bromide. A 100 bp DNA ladder (Invitrogen) (Cat. No. 15-628-019) was used as a molecular size marker.

### 2.5 Western blot analysis

Cells were treated with DMSO or sorafebnib (20 μM). Proteins were extracted from the cells using RIPA Buffer (ATTO) containing protease inhibitors and phosphatase inhibitors. Protein concentrations were determined using a Pierce BCA Protein Assay Kit (Thermo Fisher Scientific, 23227). The lysates were immediately heated for 15 min at 100 °C. The homogenates were centrifuged at 13,000g for 15 min at 4 °C, and the supernatants were collected. Cell lysates were subjected to WB analysis, as described previously [19]. 12 % SDS-PAGE gel was used for protein separation. After transferring to a nitrocellulose blotting membrane, it was blocked with 5 % skim milk at room temperature for 1 h. Membrane washing was performed by mixing 0.1 % Tween 20 in TBS-T. The primary antibodies against Grp78 (ID 3177) (Cell Signaling), ATF6 (ID 65880) (Cell Signaling), XBP1 (ID 12782) (Cell Signaling), ATF4 (ID 11815) (Cell Signaling), CHOP (ID 5554) (Cell Signaling) and SCL7A11 (ID 12691) (Cell Signaling) were diluted (1:500–1:1000) in 5 % skim milk and maintained at 4 °C overnight. Secondary antibodies were diluted (1:5000) in 5 % skim milk and bound for 1 h at room temperature. Anti-mouse IgG HRP (Cell Signaling) and anti-Rabbit IgG HRP (Cell Signaling) were used. Signal detection was carried out using enhanced chemiluminescence (ECL) substrate (Thermo Scientific Pierce ECL) and visualized on a chemiluminescence imaging system (Bio-Rad). Protein levels were quantified by using ImageJ.

### 2.6 Transfection

Cells were transfected with siRNAs using Lipofectamine RNAiMAX (Thermo Fisher Scientific) according to the manufacturer's instructions. Sorafenib treatment started 24 h after transfection. siRNAs used in this study were purchased (Thermo Fisher Scientific) (Cat. No. 4392420: s3995, s3996, and s3997).

### 2.7 Flow cytometric analyses

Lipid ROS levels were evaluated using BODIPY 581/591 C11 (Thermo Fisher Scientific, MA, USA), a fluorescent fatty acid probe that undergoes a shift in fluorescence from red to green upon oxidation by lipid-derived reactive oxygen species. Specifically, the decrease in red fluorescence (excitation/emission: 581/610 nm) was measured, serving as an indicator of lipid peroxidation. The assay was performed following previously established protocols [19,20]. In brief, cells were seeded into 6-well plates at a density of 2 × 10^5^ cells/well, cultured for more than 24 h, treated with the specified chemicals for the indicated durations, harvested, and washed twice with cold PBS. For siCHOP experiments, siCHOP were transfected for 24 h using Lipofectamine RNAiMAX transfection reagent (Thermo Fisher Scientific). They were then treated with or without sorafenib for an additional 24 h. Treated cells were harvested and washed twice with cold PBS. Cells were then stained with 2.5 μM lipid peroxidation probe (BODIPY™ 581/591 C11, Cat. No. D3861) (Invitrogen) for 30 min in the dark. Finally, cells were washed three times with PBS to remove unincorporated dyes and resuspended in 300 μL of PBS. Cells were then analyzed using a FACSCanto II flow cytometer (BD Biosciences) and FlowJo software (Ashland).

### 2.8 Public database analysis

Kaplan–Meier survival analysis was conducted to evaluate the prognostic relevance of SLC7A11 expression in kidney renal clear cell carcinoma (KIRC). Analyses were performed using publicly available datasets from the human protein atlas (https://www.proteinatlas.org), based on the cancer genome atlas (TCGA). Patients were stratified into high and low expression groups according to either the median or optimized expression cut-off values provided by each platform. Kaplan–Meier plots were used to visualize survival differences, and statistical significance was assessed using the log-rank test.

### 2.9 Statistical analysis

The data were shown as mean ± standard deviations of the means with three independent experiments. Statistical analysis was conducted through one-way analysis of variance (ANOVA) comparing the samples with their respective control using GraphPad Prism 8.0 software. Data are taken as significance when p < 0.05.

## 3 Results

### 3.1 Sorafenib induces ferroptosis in human RCC cells

**Fig. 1:**
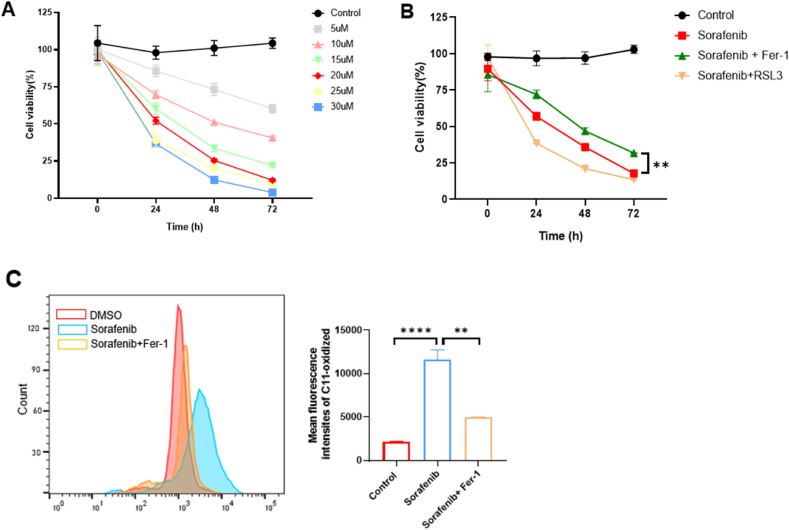
**Sorafenib induces ferroptosis in RCC.** (A) Cell viability was analyzed by CCK8 assay in A498 cells treated with DMSO (control) or various concentrations of sorafenib for 24, 48, and 72 h (n = 3/group). (B) Cell viability analyzed by CCK8 assay in A498 cells treated with DMSO (control), 20 μM sorafenib, and sorafenib in combination with a ferroptosis inhibitor (Ferrostatin-1: Fer-1) or activator (RSL3) for 24 h (n = 3/group). (C) Representative histograms of the fluorescence intensity of C11-oxidized membrane lipids in A498 cells treated with DMSO (control), 20 μM sorafenib, and sorafenib with Fer-1 for 24 h and a graph summarizing the mean fluorescence intensity of those cells (n = 3/group). ∗*p* < 0.05, ∗∗*p* < 0.01, ∗∗∗*p* < 0.001, and ∗∗∗∗*p* < 0.0001 between the indicated groups. Data are presented as the mean ± SD.

To elucidate how sorafenib mediates ferroptosis in RCC cells, we first wanted to determine its median lethal dose (LD_50_) 24 h after treating A598 RCC cells with various concentration of sorafenib. We found that sorafenib decreased cell viability in a time- and dose-dependent manner (Fig. 1A). 20 μM sorafenib resulted in approximately 50 % cell death 24 h after treatment (Fig. 1A). We used this concentration of sorafenib for all experiments. Addition of ferrostatin-1 (Fer-1), a specific ferroptosis inhibitor, partially rescued cell death caused by sorafenib, while addition of RSL3, a known ferroptosis activator, promoted sorafenib-induced cell death (Fig. 1B), in A498 cells 24 h after treatment. At this time point, it seemed that approximately 30 % of sorafenib-mediated cell death was rescued by treatment with Fer-1 (Fig. 1B). In alignment with these results, C11-BODIPY staining analysis showed that sorafenib markedly increased cell membrane peroxidation, a hall mark of ferroptosis, and Fer-1 abrogated a large portion of sorafenib-induced ferroptosis (Fig. 1C). These findings provide evidence that sorafenib can induce ferroptosis-mediated cell death in RCC cells.

### 3.2 Sorafenib induces ER stress in RCC cells

**Fig. 2:**
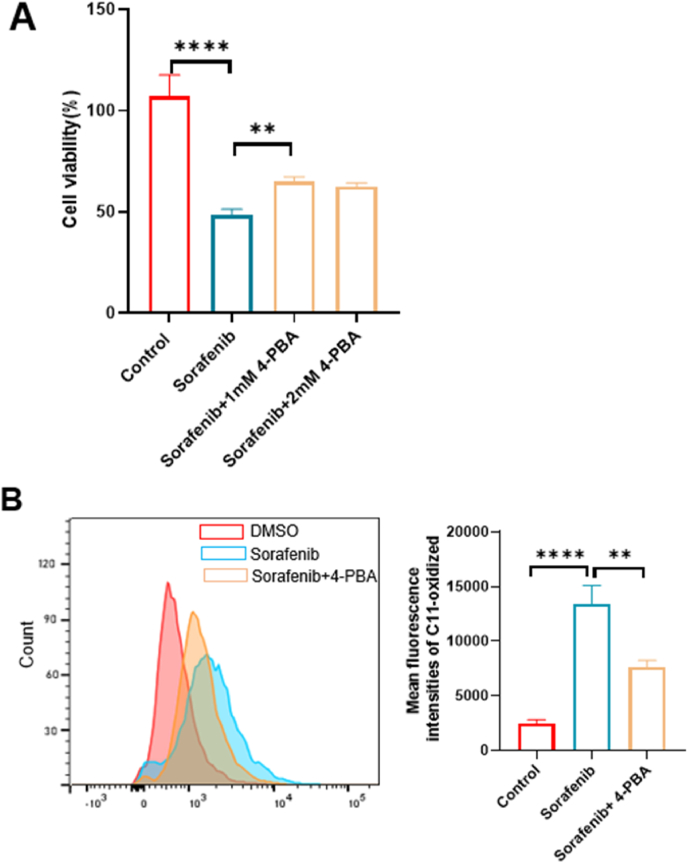
**Sorafenib induces ER stress in RCC.** (A) Cell viability was analyzed by CCK8 assay of A498 cells treated with DMSO (control), 20 μM sorafenib, and sorafenib in combination with an ER stress inhibitor for 24 h (n = 3/group). (B) Representative histograms of the fluorescence intensity of C11-oxidized membrane lipids in A498 cells treated with DMSO (control), 20 μM sorafenib, and sorafenib with an ER stress inhibitor for 24 h and a graph summarizing the mean fluorescence intensity of those cells (n = 3/group). ∗*p* < 0.05, ∗∗*p* < 0.01, ∗∗∗*p* < 0.001, and ∗∗∗∗*p* < 0.0001 between the indicated groups. Data are presented as the mean ± SD.

As ER stress contributes to lipid peroxidation and cell death, we examined whether inhibition of ER stress could rescue sorafenib-induced cytotoxicity and oxidative damage in RCC cells. Addition of the ER stress inhibitor 4-PBA partially (approximately 30 %) restored cell viability in A498 cells after exposure to sorafenib (Fig. 2A). Accordingly, sorafenib-mediated ferroptosis was partially but significantly recovered by such treatment (Fig. 2B). Notably, our results demonstrate that inhibition of ER stress with 4-PBA significantly reduced sorafenib-induced lipid peroxidation and partially restored cell viability, the recovery was not complete even with a higher concentration of 2 mM 4-PBA. This suggests that although ferroptosis is a major mode of cell death triggered by sorafenib, additional stress responses or cell death pathways might be concurrently activated. For instance, sorafenib has been reported to induce apoptosis or other non-ferroptotic mechanisms under certain conditions. Moreover, a threshold level of oxidative damage might remain even after ER stress suppression, which continues to drive ferroptotic death. Therefore, these findings support the central role of ferroptosis in sorafenib-induced cytotoxicity while also pointing to a more complex, multifactorial cellular response. Taken together, these results suggest that ER stress is linked to ferroptosis in RCC cells.

### 3.3 Sorafenib upregulates ATF4 and CHOP in RCC cells

**Fig. 3:**
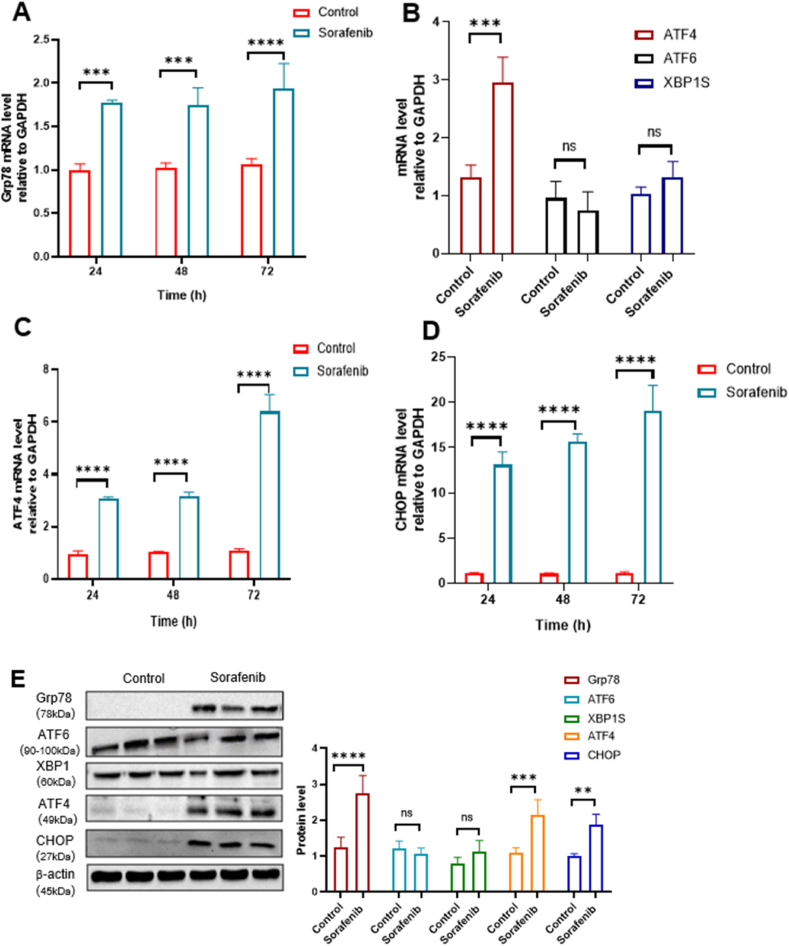
**Sorafenib upregulates ATF4 and CHOP in RCC.** (A) Relative GRP78 mRNA levels were quantified by RT-qPCR in A498 cells treated with DMSO or 20 μM sorafenib for 24, 48 and 72 h (n = 3/group). (B) Relative mRNA levels of ATF4, ATF6, and XBP1s were quantified by RT-qPCR in A498 cells treated with DMSO or 20 μM sorafenib for 24 h (n = 3/group). (C and D) Relative mRNA levels of ATF4 (C) and CHOP (D) were quantified by RT-qPCR in A498 cells treated with DMSO or 20 μM sorafenib at different time points (n = 3/group). (E) Grp78, ATF6, XBP1, ATF4 and CHOP protein levels were analyzed by Western blot in A498 cells treated with DMSO or 20 μM sorafenib for 24 h (n = 3/group). The intensity of bands was quantified using ImageJ. ∗*p* < 0.05, ∗∗*p* < 0.01, ∗∗∗*p* < 0.001, and ∗∗∗∗*p* < 0.0001 between the indicated group. Data are presented as the mean ± SD.

Treating A498 cells with sorafenib upregulated the mRNA expression levels of GRP78 (Glucose-regulated protein 78; also known as BiP) that is an important molecular regulator for the ER stress response, and its expression levels were sustained for a long time (Fig. 3A). Interestingly, sorafenib was specific in increasing the transcription of ATF4 (Activating transcription factor 4), but not of ATF6 (Activating transcription factor 6) and XBP1s (Spliced X-box binding protein 1) (Fig. 3B), suggesting that sorafenib-induced ER stress occurs mainly through the PERK-ATF4 axis. Like GRP78, the expression of ATF4 and its target gene CHOP displayed a persistent expression pattern (Fig. 3C and D). We also confirmed that their protein levels increased in A498 cells 24 h after treatment with sorafenib (Fig. 3E and F). Therefore, these results suggest that sorafenib induces persistent ER stress primarily through the ATF4-CHOP signaling pathway in RCC cells.

### 3.4 CHOP contributes to sorafenib-induced ferroptosis through inhibition of SLC7A11 in RCC cells

**Fig. 4:**
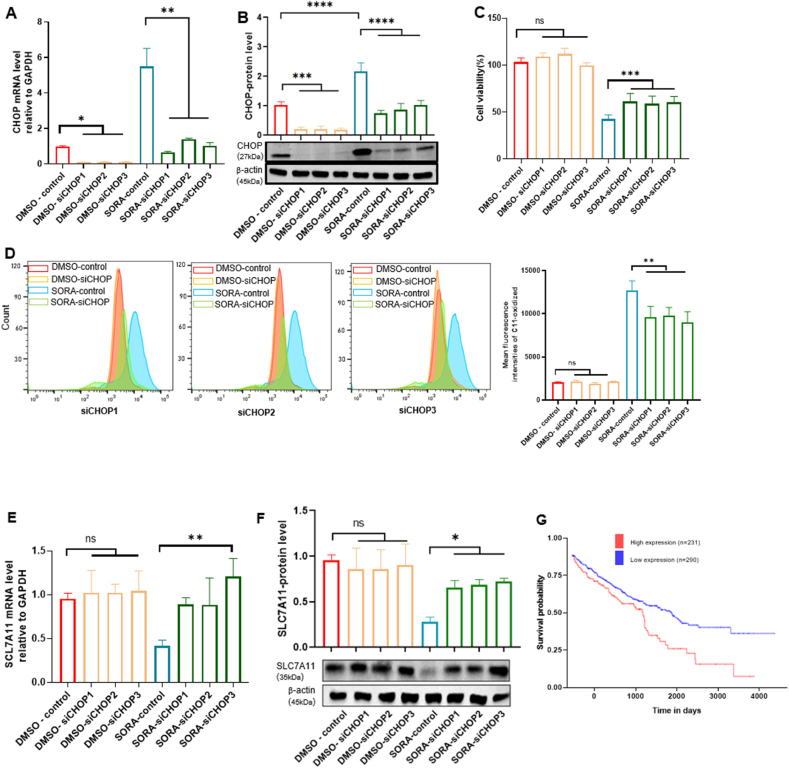
**CHOP contributes to sorafenib-induced ferroptosis through inhibition of SLC7A11 in RCC.** (A) Relative CHOP mRNA levels were quantified by RT-qPCR in CHOP siRNA knockdown A498 cells, followed by treatment with DMSO or sorafenib (SORA) 24 h after siRNA transfection (n = 3/group). (B) CHOP protein levels were analyzed by Western blot in A498 cells treated with DMSO or sorafenib with or without CHOP knockdown (n = 3/group). (C) Cell viability was analyzed by CCK8 assay in A498 cells treated with DMSO or sorafenib with or without CHOP knockdown (n = 3/group). (D) Representative histograms of the fluorescence intensity of C11-oxidized membrane lipids in A498 cells treated with DMSO or sorafenib with or without CHOP knockdown and a graph summarizing the mean fluorescence intensity of those cells (n = 3/group). (E) Relative mRNA levels of SLC7A11 were quantified by RT-qPCR in A498 cells treated with DMSO or sorafenib with or without CHOP knockdown (n = 3/group). (F) SLC7A11 protein levels were analyzed by Western blot in A498 cells treated with DMSO or sorafenib with or without CHOP knockdown (n = 3/group). (G) Kaplan-Meier survival curves of overall survival according to SLC7A11 expression in the TCGA renal clear cell carcinoma cohorts. ∗*p* < 0.05, ∗∗*p* < 0.01, ∗∗∗*p* < 0.001, and ∗∗∗∗*p* < 0.0001 between the indicated groups. Data are presented as the mean ± SD.

To evaluate the role of CHOP in sorafenib-induced cell death in RCC, we performed CHOP knockdown experiments using three individual siRNAs (siCHOP-1, siCHOP-2, and siCHOP-3). All the three siRNAs for CHOP were effective at suppressing CHOP at either a mRNA or a protein level (Fig. 4A and B). Their transfection significantly recovered cell viability in A498 cells to a similar extent after treatment with sorafenib (Fig. 4C). However, such transfection did not affect cell viability in DMSO-treated A498 cells (Fig. 4C), indicating that specific inhibition of CHOP can block sorafenib-induced cell death. Notably, CHOP knockdown significantly reduced the level of lipid peroxidation, as evidenced by the change in C11-BODIPY fluorescence intensity (Fig. 4D). In aggregate, these results suggest that CHOP plays an important role in sorafenib-induced ferroptosis in RCC.

SLC7A11 is a key protein that prevents ferroptosis. It has not been reported yet that CHOP regulates the expression of these genes. Since our data showed that treatment with sorafenib increased the expression of CHOP in A498 cells (Fig. 3D), we examined if sorafenib can inhibit the expression of SLC7A11. Indeed, sorafenib was effective at inhibiting the expression of SLC7A11 (Fig. 4E and F). However, CHOP knockdown in A498 cells restored the expression of SLC7A11 to the level of DMSO-treated A498 cells (Fig. 4E). Analysis of TCGA cohorts showed that high SLC7A11 expression was correlated with an inferior overall survival in renal clear cell carcinoma (Fig. 4G). Taken together, these results suggest that sorafenib may lead to ferroptosis in RCC by inhibiting the expression of SLC7A11 and thereby increasing oxidative stress responses.

## 4 Discussion

In this study, we discovered that sorafenib triggered ferroptosis-induced cell death in RCC cells through activation of ER stress involving the transcription factor CHOP. We first demonstrated that sorafenib significantly reduced RCC cell viability in a dose-dependent manner, an effect that was significantly reversed by the ferroptosis inhibitor Fer-1 and enhanced by the ferroptosis inducer RSL3 (Fig. 1A and B) [21,22]. Accumulation of lipid reactive oxygen species, a hallmark of ferroptosis-induced cell death, was significantly increased after sorafenib exposure and reduced upon inhibition of ferroptosis-induced cell death (Fig. 1C), suggesting that ferroptosis-induced cell death is an important mode of cell death caused by sorafenib [23]. Next, we showed that the use of 4-PBA, an ER stress inhibitor, significantly rescued cell viability and abolished lipid ROS production in sorafenib-treated RCC cells (Fig. 2A and B), revealing a causal role of ER stress in inducing ferroptosis [22]. Notably, sorafenib increased markers of endogenous ER stress such as ATF4 and CHOP at both the transcript and protein levels (Fig. 3A and F). Interestingly, several sorafenib-like drugs, such as sunitinib, regorafenib, and lapatinib, have also been reported to trigger ferroptosis through endoplasmic reticulum (ER) stress signaling pathways, including activation of the ATF4–CHOP axis. For example, regorafenib was shown to promote ferroptosis in hepatocellular carcinoma by upregulating ATF4 and CHOP expression, similar to sorafenib's mechanism of action [24]. Likewise, lapatinib has been found to sensitize breast cancer cells to ferroptosis via ATF4-dependent transcriptional responses [25]. These findings support the notion that the ATF4–10.13039/100006458CHOP pathway may be a core module for ferroptotic cell death in cancer cells that multikinase inhibitors share. While ER stress is widely involved in apoptosis and autophagy [26], its direct contribution to ferroptosis-induced cell death signaling remains unexplored. Our data support the premise that ER stress, rather than acting solely as a stress response, actively mediates ferroptosis-induced cell death in this context.

Silencing of CHOP by siRNA significantly reduced sorafenib-induced cytotoxicity and lipid peroxidation (Fig. 4C and D), suggesting that CHOP is indispensable for the execution of ferroptosis-induced cell death during sorafenib treatment. Notably, CHOP depletion resulted in the upregulation of SLC7A11 after sorafenib treatment (Fig. 4E, F, and 4G) - an important regulator of cellular redox balance and resistance to ferroptosis, suggesting that CHOP may regulate ferroptosis mainly through a transcriptional mechanism affecting antioxidant pathways. Taken together, our findings establish an intriguing mechanistic framework in which sorafenib induces ER stress in RCC cells, thereby activating the ATF4–CHOP axis, which in turn facilitates ferroptosis through dysregulation of ROS lipid metabolism. This integrated stress response may serve as a molecular switch that determines cell fate under conditions of metabolic or therapeutic challenge [27]. It is conceivable that CHOP, under persistent stress, reprograms gene expression in a manner that inhibits adaptive antioxidant responses while promoting iron-dependent lipid peroxidation. However, it remains unclear how sorafenib and other kinases inhibitors specifically activated the ATF4-CHOP axis of ER stress. In that there is an increase in ATF4 mRNA and protein levels in response to sorafenib (Fig. 3B, C, and 3E), it is likely that sorafenib targets signaling upstream of ATF4, including PERK and eIF2Aα. This is an urgent issue to be solved in the future.

Translationally, our results have important therapeutic implications. The identification of ER stress and CHOP as important regulators of ferroptosis provides novel targets for combination therapy. Agents that enhance ER stress or inhibit anti-ferroptosis pathways may enhance the efficacy of sorafenib, while selectively reducing maladaptive ER stress may reduce off-target toxicity. However, this study has inherent limitations. The lack of in vivo validation and clinical sample analysis limits the generalizability of our findings. Future work involving patient-derived genetic and xenograft models will be needed to confirm the translational relevance of the ER stress–CHOP–ferroptosis axis in RCC.

## CRediT authorship contribution statement

**Dinh Thi Nhung:** Writing – original draft, Investigation, Formal analysis, Conceptualization. **Obadah E.A. Yousif:** Data curation. **Byungsuk Kwon:** Writing – review & editing, Supervision, Funding acquisition, Conceptualization.

## Declaration of AI-assisted technologies in the writing process

The authors received ChatGPT help in writing the manuscript.

## Funding

This work was supported by the 10.13039/501100002568University of Ulsan Research Fund (2024-0367).

## Declaration of competing interest

The authors declare that they have no known competing financial interests or personal relationships that could have appeared to influence the work reported in this paper.
